# Neutrophil Extracellular Traps Affect Human Inner Ear Vascular Permeability

**DOI:** 10.3390/ijms25189766

**Published:** 2024-09-10

**Authors:** Marijana Sekulic, Stavros Giaglis, Nina Chatelain, Daniel Bodmer, Vesna Petkovic

**Affiliations:** 1 Department of Biomedicine, University Hospital Basel, University of Basel, 4031 Basel, Switzerland; stavros.giaglis@unibas.ch (S.G.); nina.chatelain@unibas.ch (N.C.); daniel.bodmer@usb.ch (D.B.); vesna.petkovic@unibas.ch (V.P.); 2 Clinic for Otorhinolaryngology, University Hospital Basel, University of Basel, 4031 Basel, Switzerland

**Keywords:** blood–labyrinth barrier, hearing loss, Meniere’s disease, TEER, tissue model

## Abstract

The integrity of the blood–labyrinth barrier (BLB) is essential for inner ear homeostasis, regulating the ionic composition of endolymph and perilymph and preventing harmful substance entry. Endothelial hyperpermeability, central in inflammatory and immune responses, is managed through complex intercellular communication and molecular signaling pathways. Recent studies link BLB permeability dysregulation to auditory pathologies like acoustic trauma, autoimmune inner ear diseases, and presbycusis. Polymorphonuclear granulocytes (PMNs), or neutrophils, significantly modulate vascular permeability, impacting endothelial barrier properties. Neutrophil extracellular traps (NETs) are involved in diseases with autoimmune and autoinflammatory bases. The present study evaluated the impact of NETs on a BLB cellular model using a Transwell^®^ setup. Our findings revealed a concentration-dependent impact of NETs on human inner ear-derived endothelial cells. In particular, endothelial permeability markers increased, as indicated by reduced transepithelial electrical resistance, enhanced dextran permeability, and downregulated junctional gene expression (*ZO1*, *OCL*, and *CDH5*). Changes in cytoskeletal architecture were also observed. These preliminary results pave the way for further research into the potential involvement of NETs in BLB impairment and implications for auditory disorders.

## 1. Introduction

Endothelial hyperpermeability is increasingly recognized as both a pivotal cause and a consequence of inflammatory and immune responses [1]. The endothelium, which lines the inside of all blood vessels, acts as a crucial barrier between the blood and surrounding tissues. It not only forms a physical blockade preventing the unwanted leakage of blood elements but also selectively controls the movement of blood fluids and large molecules into the adjacent tissue. The term “blood–labyrinth barrier” (BLB) is used to describe the interface between the vascular system and the inner ear’s fluid compartments, specifically the endolymph and perilymph [2,3]. The BLB in the stria vascularis consists of vascular endothelial cells (ECs) surrounded by basement membrane, pericytes (PCs), and perivascular-resident macrophage-like melanocytes. This barrier is essential in maintaining the ionic balance of inner ear fluids and safeguarding the inner ear from harmful substances. Investigations into the transport of dyes and pharmaceutical compounds from the systemic circulation to the inner ear fluids have revealed the selective nature of the BLB. These studies have also demonstrated that the BLB effectively regulates the composition of inner ear fluids, ensuring that it is distinct from the composition of the blood and other bodily fluids, such as the cerebrospinal fluid [4]. ECs lining the internal surface of blood vessels are connected by tight junctions and form a crucial interface between the circulating blood and the vessel wall. An understanding of the dynamics of the BLB and specifically its endothelial layer is critical for the development of targeted therapeutics and their delivery to the inner ear. Ideally, these candidates would modulate the BLB’s inflammatory response by inhibiting or enhancing it. 

Recent research has linked disruptions in the BLB to various inner ear disorders, including acoustic trauma, autoimmune inner ear disease, and presbycusis [5,6,7]. Although the BLB has been implicated in Meniere’s disease, studies investigating the ultrastructure of the BLB in people with Meniere’s disease and unaffected controls are lacking. The causes and mechanisms of this disease, characterized by fluctuating hearing loss, recurrent vertigo, and a sensation of ear fullness, remain unclear. This gap in understanding underscores the need for further investigation of the BLB in Meniere’s disease and similar inner ear pathologies.

Among the various leukocyte subtypes present in the bloodstream, polymorphonuclear granulocytes (PMNs), commonly known as neutrophils, play a significant role in vascular permeability. These cells affect the properties of the endothelial barrier through direct interactions, including adhesion and transmigration, and by secretion of bioactive compounds that can disrupt the integrity of the barrier [8]. PMNs are key cells in the innate immune response and combat infections primarily through mechanisms like phagocytosis and degranulation. When activated, PMNs also can expel neutrophil extracellular traps (NETs) in reaction to various stimuli. NETs consist of intricate networks of cell-free DNA, histones, and proteins from PMN granules, including neutrophil elastase, cathepsin G, and myeloperoxidase [9]. These structures have been implicated in a wide array of health conditions, including cardiovascular, inflammatory, and autoimmune diseases and metabolic disorders [10,11,12]. The involvement of NETs in these conditions is increasingly recognized as a significant factor in the severity and outcomes of these diseases [12,13]. 

Here, we investigated how NETs affect the human-derived inner ear BLB using a representative Transwell^®^ system-based model and explored the potential implications for certain BLB-related inner ear pathologies.

## 2. Results

### 2.1. Dose-Dependent NET-Induced Endothelial Toxicity

**Figure 1:**
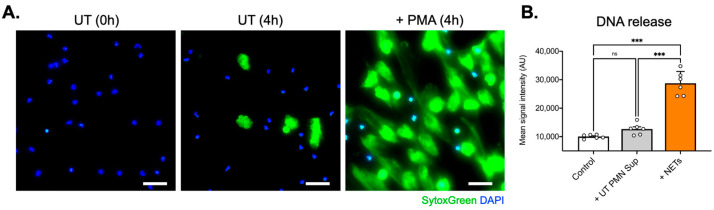
NET generation from healthy donors’ PMNs. (**A**) SytoxGreen and DAPI fluorescence captions depicting extracellular DNA and cell nuclei, respectively. (**B**) Spectro-photometrical determination of DNA quantities (NETs) in cell supernatants. Magnification: 10×; scale bars: 100 μm; ns: not significant; ***: *p* < 0.001; data are presented as the mean ± SD.

NETs were generated in vitro from healthy donors’ PMNs after a 4 h culture at 37 °C, stimulated with phorbol-12-myristate-13-acetate (PMA; 140 nM, Sigma-Aldrich , Burlington, MA, USA), and collected as culture supernatants; extracellular traps also were collected from untreated PMNs that had undergone spontaneous NET formation (non-PMA-treated PMN supernatants—UT PMN Sup) (Figure 1A). NETs were quantified using the DNA-binding dye SytoxGreen™ (5 μM, Invitrogen, Waltham, MA, USA) in a fluorescence microplate reader (Figure 1B). These supernatants were utilized in the subsequent incubation experiments with human inner ear-derived endothelial cells (ECs).

**Figure 2:**
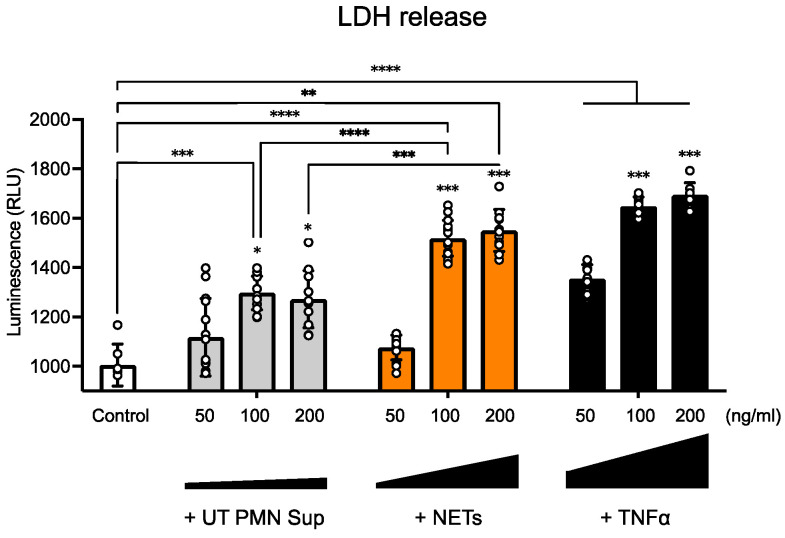
NETs induce endothelial toxicity in a dose-dependent manner. An LDH test was performed after 96 h of exposure to 50 ng/mL, 100 ng/mL, and 200 ng/mL of untreated PMN supernatant (UT PMN Sup) and NETs, with TNFα as a positive control. All three treatments showed increased luminescence intensity, indicating increased cellular toxicity compared with the control, with TNFα causing the most significant impact and the UT PMN supernatant having the lowest impact. At 100 ng/mL, NETs showed a significant increase in luminescence compared with both the EC medium-only control and the 100 ng/mL UT PMN supernatant. With the 200 ng/mL treatment, the cytotoxic luminescence signal increased significantly for NETs and TNFα, whereas UT PMN supernatant treatment values remained similar to values obtained with a 100 ng/mL concentration. Asterisks above the 100 ng/mL and 200 ng/mL bars indicate comparisons to the respective 50 ng/mL treatments. *: *p* < 0.05, **: *p* < 0.01, ***: *p* < 0.001, ****: *p* < 0.0001. Total number of individual treatment experiments (*n* = 3); data are presented as the mean ± SD.

To test the potential endothelial toxicity of NETs, we exposed human inner ear–derived endothelial cells (ECs) to several concentrations of NETs and compared them to supernatants from non-PMA-treated PMNs (UT PMN Sup) and to tumor necrosis factor alpha (TNFα), which is known to be toxic to ECs [14], as well as controls exposed to EC medium only. To assess the effects of these exposures on the ECs, we used the lactate dehydrogenase (LDH) assay, which measures LDH release from the cytoplasm into the medium, providing an accurate reflection of cell viability in vitro. At 50 ng/mL, luminescence was not significantly elevated by any of the treatments except for TNFα, which was considered a positive control. However, at 100 ng/mL, ECs treated with NETs showed a significant increase in luminescence compared with both the EC medium-only control and the 100 ng/mL UT PMN supernatant. The 100 ng/mL TNFα treatment also resulted in a significant increase in luminescence compared to the EC medium-only control, as expected. At 200 ng/mL, NETs caused a significant increase in cytotoxic luminescence compared with both the control and the 200 ng/mL UT PMN supernatant. Similarly, the 200 ng/mL TNFα treatment produced a significant increase in cytotoxicity compared with the EC medium-only control, as expected. Additionally, at concentrations of 100 ng/mL and 200 ng/mL, ECs treated with UT PMN supernatant also showed a slight increase in cytotoxicity compared with the control (Figure 2). Overall, a dose-dependent increase in luminescence intensity was observed for all three agents, indicating cellular toxicity.

### 2.2. Endothelial Monolayer Integrity Changes upon NET Exposure

**Figure 3:**
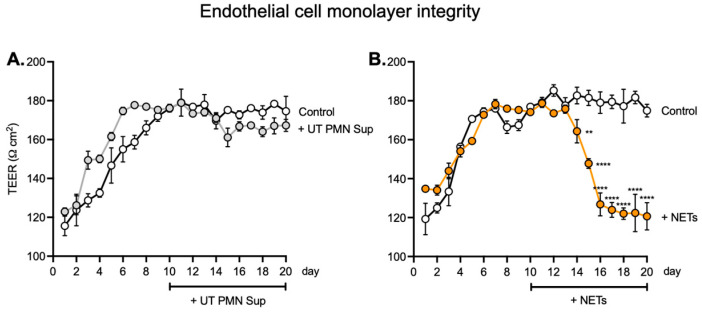
TEER values are altered in the endothelial monolayer upon NET exposure. (**A**) TEER values after seeding human ECs on a Transwell^®^ insert with a polyester membrane. Treatment with 100 ng/mL non-PMA-treated PMN supernatant (UT PMN Sup) started on day 10 and continued over the next 10 days. TEER measurements were performed once per day, with the first measurement starting 24 h after seeding and labeled as day 1. TEER measurement was performed in three consecutive repetitions for each individual well. Control cells were treated with media only. No significant difference between the UT PMN supernatant and control conditions was observed during the treatment period. (**B**) TEER values after treatment with NETs (100 ng/mL) vs. control showed a significant drop, starting from day 14 and continuing until the end of the treatment period. **: *p* < 0.01; ****: *p* < 0.0001; number of individual experiments with all treatment conditions (*n* = 6). Data are presented as the mean ± SD.

To evaluate monolayer integrity, a main characteristic of the EC layer in the blood vessel barrier, we used transepithelial/transendothelial electrical resistance (TEER), which represents the golden standard evaluation methodology. Measurements were performed on human ECs seeded on the luminal cells and PCs on the abluminal side of Transwell^®^ inserts with a polyester membrane. Treatment with NETs (100 ng/mL) was initiated on day 10 and continued for the next 10 days, and TEER measurements were performed once daily, with the first measurement starting 24 h after seeding and labeled as day 1 (Figure 3). TEER measurement was performed by three consecutive repetitions for each individual Transwell^®^. Control cells were treated with vehicle only. No significant difference was observed between the UT PMN supernatant and controls during the treatment period (Figure 3A). By contrast, TEER values after incubation with 100 ng/mL NETs showed a marked drop after treatment compared with the control, starting from day 14 and continuing until the end of the treatment period (Figure 3B). This clear decrease in TEER values indicated a significant disruption of the endothelial barrier after treatment with NETs.

### 2.3. Effects of NETs on Junctional Gene Expression, Cytoarchitecture, and Permeability

**Figure 4:**
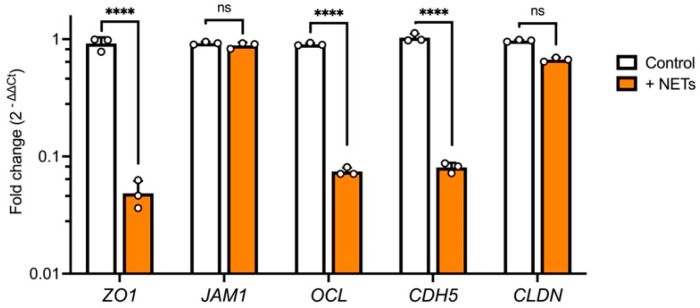
Expression of junctional genes is altered under the influence of NETs. Treatment with NETs (100 ng/mL) for 96 h resulted in a significant decrease in *ZO1*, *OCL*, and *CDH5* expression, whereas JAM1 and CLDN levels remained unchanged compared with the control. Data were obtained from six donor-derived cell cultures and performed in triplicate (technical); number of individual experiments (*n* = 3); ns: not significant; ****: *p* < 0.0001; data are presented as the mean ± SD.

Given the decrease in TEER values with exposure to NETs, indicating the disruption of the barrier, we further investigated the expression of a series of major tight junction genes most responsible for endothelial barrier integrity at the transcriptional level by means of qPCR. We found that incubation with NETs resulted in a significant decrease in *ZO1*, *OCL*, and *CDH5* expression, whereas *JAM1* and *CLDN* expression levels remained stable compared with the control (Figure 4).

**Figure 5:**
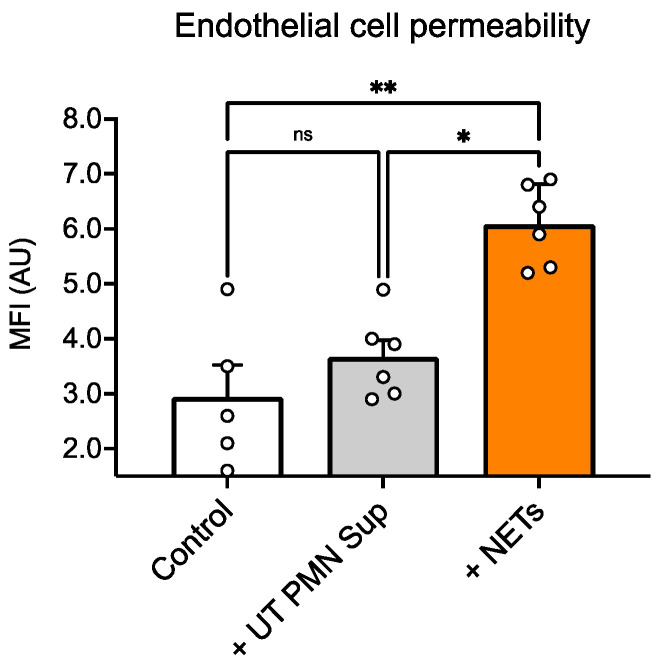
Increased EC permeability in the presence of NETs. Incubation of the endothelial cells with NETs (100 ng/mL) for 96 h produced a significant increase in dextran influx compared with the untreated cells, whereas the non-PMA-treated PMN supernatant (UT PMN Sup) did not result in any significant changes between control and treated cells. MFI: mean fluorescence intensity; ns: not significant; *: *p* < 0.05, **: *p* < 0.01; number of individual experiments with all treatment conditions (*n* = 6). Data are presented as the mean ± SD.

Furthermore, we examined permeability using 70-kDa FITC-dextran and found that treatment with NETs resulted in a significant increase in dextran influx compared with untreated wells, whereas exposure to non-PMA-treated PMN supernatant (UT PMN Sup) did not cause any significant differences compared with the control wells (Figure 5). 

**Figure 6:**
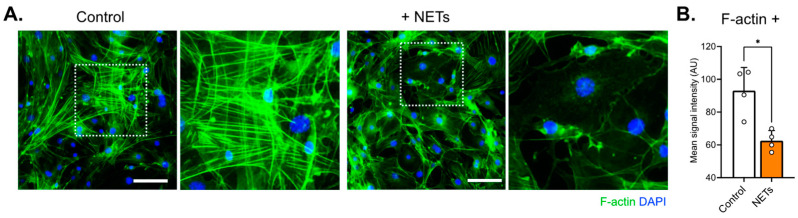
Decreased actin signal and minor rearrangement were observed in EC samples exposed to NETs. (**A**) Cells were stained with phalloidin conjugated with Alexa-488 (green) and DAPI (blue). In the NET-treated cells (100 ng/mL; right), changes in F-actin arrangement and a decrease in phalloidin intensity compared with control (left) were observed. Scale bar: 100 µm. (**B**) The fluorescence signals were weaker in NET-treated cells, as confirmed by signal quantification. *: *p* < 0.05, number of individual experiments (*n* = 3). Data are presented as the mean ± SD.

In addition, we used phalloidin green (488 nm) to stain actin filaments and observed some differences in the overall cytoarchitecture between treated and untreated cells, including a decrease in the fluorescence signal in the treated culture (Figure 6A,B). These results indicate that NETs affect endothelial barrier integrity and permeability.

## 3. Discussion

Vascular leakage is increasingly recognized for its significance in many infectious or inflammatory diseases [15], including in the inner ear. A study using post-contrast magnetic resonance imaging showed increased vascular permeability of the blood–perilymph barrier in the cochlea of patients with Meniere’s disease and significantly increased permeability compared with patients with sudden sensorineural hearing loss [16]. A recent research focus has been protecting and repairing endothelial barriers in blood vessels affected by these conditions, and various molecules have shown potential in improving these barriers [1,17,18]. Although these molecules are effective in animal studies, many have yet to prove effective in clinical settings. For this reason, we focused on creating a human cell-derived in vitro model that could recapitulate events within the cochlear BLB under various treatment conditions. 

In previous work, we tested a Transwell^®^ model in which we co-cultured human stria vascularis-derived primary ECs and PCs on each side of the porous membrane; exposed the ECs to TNF-α, interleukin-6, and lipopolysaccharide; and observed their influence on junctional proteins and endothelial permeability. The results indicated the development of an inflammatory environment that affected BLB permeability and modelled an inflammatory state within the stria vascularis [19]. PMNs regulate endothelial permeability by altering the structure and function of cell–cell junctions, the glycocalyx, and focal adhesions. These cells cause related barrier dysfunction by producing reactive oxygen species, secreting inflammatory mediators, and releasing granular contents, and NET production can be induced by similar factors [20,21]. 

In the current study, we thus investigated the potential effect of NETs on the inner ear endothelial barrier, using a human cell-derived model of the inner ear vascular barrier as an alternative to animal models and a more physiologically relevant approach for human applications. We first found that at several tested concentrations, UT PMN supernatant and NETs showed increased cellular toxicity compared with the control at 96 h of exposure, as measured by the LDH release into the medium. NETs also showed a steady dose-dependent increase in cellular toxicity (Figure 2). This result is in line with previous in vivo findings of EC damage by various PMN-related mechanisms [22,23]. We also used TNFα as a known toxic substance for the ECs, which produced a significant cytotoxicity increase at all three concentrations tested [14,24,25,26]. To assess the effects on the endothelial monolayer of longer NET exposure, we expanded exposure time to 10 days for the concentration of 100 ng/mL. After seeding human ECs, we monitored TEER as a measure of barrier integrity. The values became stable around day 7, and the 100 ng/mL treatment started on day 10 and continued over the next 10 days. We observed no significant difference between UT PMN supernatant and controls during the treatment period, but TEER values after 100 ng/mL NET treatment vs. controls declined significantly from day 14 and continued to decrease until the end of the treatment period. These results indicated a lack of barrier integrity after treatment with NETs (Figure 3) and are in keeping with previously described results for the impact of NETs on the EC barrier [27,28]. 

To further evaluate these findings, we tested the expression of major junctional genes. NET treatment resulted in a significant decrease in *ZO1*, *OCL*, and *CDH5* expression, whereas *JAM1* and *CLDN* levels remained unchanged compared with the control (Figure 4). These results are similar to previously reported findings for epithelial and endothelial junction genes and proteins [29,30]. NETs have been associated with inflammation-induced oxidative stress, which was shown to cause the disassembly of adherence junctions (AJ) at cell–cell contacts, leading to increased permeability. Reactive oxygen species (ROS) also alter the structure of tight junctions (TJs), including proteins such as occludin, ZO-1, and claudin-5 [31]. These alterations include the downregulation of protein expression, a shift from membrane to cytoplasmic localization, and a decrease in TJ barrier tightness. The mechanisms by which ROS disrupt the endothelial barrier are well-documented. ROS can compromise barrier integrity either by directly damaging structural components like AJs, TJs, and actin filaments or by indirectly activating intracellular signaling pathways that regulate endothelial barrier function [32]. This is particularly interesting for inner ear diseases with underlying inflammatory and oxidative stress causes.

We also performed a functional permeability assay with dextran and found that NET treatment resulted in a significant increase in dextran influx compared with untreated ECs, whereas treatment with UT PMN supernatant resulted in no significant changes between control and treatment wells (Figure 5). Interestingly, we observed that the UT PMN supernatant, at the same concentration, showed an increased toxicity signal, as indicated by LDH release, compared with the control. However, there were no changes in permeability under the same test conditions. The UT PMN supernatant used in this study was obtained from non-treated, spontaneously activated PMNs—possibly through autophagy-driven mechanisms—as shown in Figure 1. Despite the cellular toxicity caused by these PMNs, the damage appears insufficient to significantly compromise the endothelial barrier, as no increase in permeability was observed. The results of this study suggest that only a substantial increase in the presence of NETs significantly affects endothelial barrier properties. In addition, it has been shown that PMN adhesion initiates several intracellular processes within the endothelium, resulting in increased paracellular endothelial permeability. The regulation of biochemical signal transduction at these junctions, as well as cell–cell communication, involves both phosphorylation and S-nitrosylation. These mechanisms have been shown to be critical in the endothelial response to PMN adhesion, which ultimately increases paracellular permeability [33,34,35].

These findings suggest that NETs play a role in increasing vascular permeability in the inner ear, in keeping with previous reports that NETs increase albumin or 10 kDa dextran influx across EC monolayers through junctional disruption [36,37,38]. One possible mechanism could involve serine proteinases and matrix metalloproteinases, which are enriched in NETs and can cleave vascular endothelial–cadherin connections and compromise junction integrity [39,40]. In addition, actin immunostaining in the current work revealed decreased signal intensity and different cytoarchitecture arrangement in cells treated with NETs compared with control cells (Figure 6). Similarly, actin rearrangements have been described in human pulmonary artery ECs exposed to NETs [30].

In this study, we initiated the exploration of the detrimental effects of NETs on the endothelial barrier in the inner ear. Evidence from existing research on NETs indicates that endothelial damage is predominantly mediated by inflammation-induced oxidative stress. The direct cytotoxic effects of NET components, coupled with the inflammatory milieu and oxidative stress, contribute to the degradation of the glycocalyx on endothelial cells. This degradation leads to increased endothelial permeability through junctional disruption, the upregulation of adhesion molecules, and the induction of apoptosis [41]. Additionally, activated endothelial cells with enhanced glycolytic activity may further amplify inflammation and oxidative stress [42].

Our current findings may have implications for clarifying pathologies related to altered vascular permeability in hearing loss and Meniere’s disease in particular. NET-triggered cytokine cascades are implicated in a series of diseases arising from autoimmune and autoinflammatory biological mechanisms [43,44]. For instance, matrix metalloproteinases originating from NETs could further activate barrier-disrupting cytokines and chemokines. Indeed, Meniere’s disease appears to be linked to cytokine-related autoinflammation, eventually in association with the formation of NETs and elevated cytokine levels in the circulation [45]. One of the limitations of this study is the lack of patient samples, which restricts our ability to directly evaluate how NETs from patients affect the endothelial barrier. This also limits our capacity to explore the specific mechanisms by which NETs contribute to endothelial dysfunction in a clinical context. The inclusion of patient-derived samples would provide a more accurate representation of the pathological processes and could help validate our findings in a real-world setting. Further studies are necessary to confirm the potential role of NETs in Meniere’s disease. 

In conclusion, our study demonstrates that NETs significantly increase vascular permeability in the inner ear barrier model by disrupting endothelial junctions and cytoskeletal structures. NETs, unlike unstimulated PMNs, caused a significant increase in cytotoxicity, reduced TEER values, altered junctional protein expression, and increased dextran influx. These findings suggest a potential role for NETs in the pathogenesis of hearing loss and Meniere’s disease, particularly in inflammation-induced vascular permeability. Our future research will focus on analyzing liquid biopsies and PMNs from patients with Meniere’s using our human cell-based model to further explore the impact of NETs on endothelial cells and identify permeability modifiers that could serve in this context as potential therapeutic candidates.

## 4. Materials and Methods

### 4.1. Human Tissue Collection, Cell Isolation, and Cell Culture 

We established a protocol for the isolation, maintenance, and differentiation of human stria vascularis cells from human post-mortem tissues obtained from the Pathology Institute in Basel, Switzerland (ethical permit: EKNZ 2020-01379). Autopsy-derived post-mortem human temporal bones were used as a tissue source due to the paucity of surgical procedures that allow for the acquisition of healthy stria vascularis tissue. The deceased donors ranged in age from 50 to 75 years; one was female, and five were male. Briefly, the healthy tissue section was removed (method fully described in our previous publication [12]), and samples were placed in a human EC or PC medium for transport (ScienCell, Carlsbad, CA, USA, cat# 1001 and cat# 1201). Of note, instead of using the supplied fetal bovine serum, we used human serum (Sigma-Aldrich, Burlington, MA, USA, cat# H3667). Immediately upon collection, the tissue was transported to the lab for further processing. Samples were cut into smaller pieces and trypsinized with 0.25% trypsin (Sigma-Aldrich, Burlington, MA, USA, cat# T4049) for 5 min, followed by the addition of soybean trypsin inhibitor (Defined Trypsin Inhibitor, Gibco, Grand Island, NY, USA cat# R007100) as an animal-free alternative to blocking solution containing fetal bovine serum. The tissue pieces were then vigorously pipetted at least 30× to allow the loosening up of the cells, and the whole suspension was centrifuged at 1100 rpm for 10 min. After the supernatant was removed, cells were resuspended in an appropriate amount of the medium and transferred to a 24-well plate coated with human fibronectin for EC cells and poly-l-lysin for PC cells (Sigma-Aldrich, Burlington, MA, USA, cat# F0895). 

### 4.2. PMN Isolation and NET Generation

PMNs were negatively selected from EDTA–human whole blood samples using the EasySep™ Direct Human Neutrophil Isolation Kit (StemCell Technologies, Vancouver, BC, Canada, cat# 19666). Samples were collected within a volume range of 1–5 mL, and the whole blood sample was added to a 14 mL polystyrene round-bottom tube (e.g., cat# 38008), followed by the addition of 50 μL of Isolation Cocktail per milliliter of sample. Next, 50 μL of RapidSpheres™ was added per milliliter of sample and mixed and incubated at room temperature for 5 min; EasySep™ buffer (StemCell Technologies, Vancouver, BC, Canada, cat#20144) was then added to top up to 12 mL and mixed with gentle pipetting up and down 2–3 times. The tube (without the lid) was placed into the magnet and incubated at room temperature for 10 min. With careful pipetting, the enriched cell suspension was transferred into a new 14 mL tube. Following this step, 50 μL of RapidSpheres™ was added per milliliter of sample to the new tube containing the enriched cells, followed by mixing and incubation at room temperature for 5 min. The tube was then removed from the magnet, and a new tube was placed (without the lid) into the magnet and incubated at room temperature for 5 min for a second separation. The enriched cell suspension was transferred into a new 14 mL tube, and only the clear fraction was collected. A third separation was performed, as described above, and the clear fraction was collected once more, followed by centrifugation at 300× *g* for 8 min. After the supernatant was discarded and the RPMI medium was added, cells that were ready for further stimulation were counted. 

Next, 1.5 × 10^6^ purified PMNs were seeded per well in 6-well culture plates (Greiner Bio-One) and stimulated with phorbol-12-myristate-13-acetate (100 ng/mL, 140 nM; Sigma-Aldrich , Burlington, MA, USA) for 4 h at 37 °C to trigger NET formation. After the careful removal of the medium and cautious washing with RPMI, an additional 2 mL of RPMI was added to each well. NETs were collected after vigorous agitation of the supernatant medium. The collected medium was centrifuged at 300× *g* for 8 min, and the supernatant phase, containing NETs, was newly collected and stored at −20 °C until further use. Extracellular DNA, as an indirect marker of NET formation, was measured using SytoxGreen™ (5 μM, Invitrogen, Waltham, MA, USA) in a fluorescence microplate reader. Extracellular traps also were collected from untreated PMNs that had undergone spontaneous NET generation (non-PMA-treated PMN supernatants—UT PMN Sup). 

### 4.3. Cell Culture and Treatment 

The cells were seeded and incubated at 37 °C in 5% CO_2_. The growth medium for ECs consisted of 500 mL of an EC basal medium, 25 mL of human serum (Sigma-Aldrich , Burlington, MA, USA, cat# H3667), 5 mL of EC growth supplement (ScienCell, Carlsbad, CA, USA, cat# 1052), and 5 mL of penicillin/streptomycin solution (ScienCell, Carlsbad, CA, USA, cat# 0503). The PC growth medium consisted of 500 mL of PC basal medium, 10 mL of human serum (Sigma-Aldrich , Burlington, MA, USA, cat# H3667), 5 mL of PC growth supplement (ScienCell, Carlsbad, CA, USA, cat# 1252), and 5 mL of penicillin/streptomycin solution (ScienCell, Carlsbad, CA, USA, cat# 0503). Before use in the experiments, cells were cultured and expanded in T25 or T75 flasks coated with appropriate attachment factors. The cells used for the experiments were from passages 2 or 3. After expansion in the flask for seeding on the Transwell^®^ plate, they were trypsinized and then resuspended in the appropriate cell medium. The medium was changed every 2 days. Before cells were placed in the Transwell^®^ plate, the cell type was validated at the gene and protein levels using immunostaining and gene expression of marker proteins. Treatments were performed with 50 ng/mL, 100 ng/mL, or 200 ng/mL of supernatants of untreated PMNs (UT PMN Sup), NETs, or tumor necrosis factor (TNF)⍺. Lactate dehydrogenase (LDH), gene expression, and dextran assays were assessed after 96 h of culture, while the transepithelial electrical resistance (TEER) assay was followed for 10 days. Controls were incubated with the medium only.

### 4.4. LDH Assay

The LDH-Glo™ Cytotoxicity Assay (Promega, Madison, WI, USA, cat#J2381) was used to measure LDH released from membrane-damaged cells and assess cytotoxicity. ECs were treated with 50 ng/mL, 100 ng/mL, or 200 ng/mL of UT PMN supernatant, NETs, or TNF⍺ for 96 h each. Samples were processed according to the manufacturer’s protocol. Briefly, 2.5 µL of the cell treatment/control medium was mixed with 47.5 µL of LDH Storage Buffer in a 96-well plate (Corning Costar^®^, Corning, NY, USA, cat# 3917), followed by the addition of 50 µL of LDH detection reagent mix and left to incubate for 60 min at room temperature. Following incubation, luminescence was read on a plate reader (BioTek, Winooski, VT, USA, Synergy H1).

### 4.5. qPCR

RNA was isolated from collected cells and extracted using the Direct-Zol RNA MiniPrep Kit (Zymo Research, Irvine, CA, USA, cat#R2050) according to the manufacturer’s instructions. Total RNA (1000 ng) was reverse-transcribed using a High-Capacity cDNA Reverse Transcription Kit (Applied Biosystems, Waltham, MA, USA). We analyzed triplicate samples by quantitative (q)PCR on an ABI Prism 7900HT Sequence Detection System (Applied Biosystems) using the Power SYBR Green Master Mix (Applied Biosystems, Applied Biosystems, Waltham, MA, USA). Primers targeting GAPDH were synthesized by Microsynth (Balgach, Switzerland) and added at a final concentration of 250 nM per reaction. The full primer sequences used in this study were as follows (all 5’–3’): *TJP1* (*ZO1*): forward, CAA CAT ACA GTG ACG CTT CAC A and reverse, CAC TAT TGA CGT TTC CCC ACT C; *F11R* (*JAM1*): forward, ATG GGG ACA AAG GCG CAA G and reverse, CAA TGC CAG GGA GCA CAA CA; *OCLN* (*OCL*): forward, ACA AGC GGT TTT ATC CAG AGT C and reverse, GTC ATC CAC AGG CGA AGT TAA T; *CDH5*: forward, TTG GAA CCA GAT GCA CAT TGA T and reverse, TCT TGC GAC TCA CGC TTG AC; *CLDN5*: forward, CTC TGC TGG TTC GCC AAC AT and reverse, CAG CTC GTA CTT CTG CGA CA; *GAPDH*: forward, GGA GCG AGA TCC CTC CAA AAT and reverse, GGC TGT TGT CAT ACT TCT CAT GG. The relative quantities of specifically amplified cDNAs were calculated by the comparative threshold cycle method (2^–∆∆Ct^), and GAPDH expression was used as the endogenous reference.

### 4.6. TEER 

TEER was measured with a Voltohmmeter EVOM3. After cells were counted using Trypan Blue staining in an automated cell counter (Bio-Rad, Hercules, CA, USA, TC20), the cell suspension was placed on each side of the coated Transwell^®^ membrane in two steps. First, the inserts were flipped upside down, and 150 μL of PC suspension was placed on the abluminal side. The inserts were then returned to the incubator for 3 h to allow cells to settle and attach. In the second step, after 3 h, the inserts were flipped back into their original position and placed in the PC medium-containing 24-well slot. The EC suspension was added on the luminal side of the insert, and cells were left for 3 h to attach in the small volume of 150 μL before the rest of the EC medium was added.

For TEER measurements, ECs were seeded at a density of 2 × 10^5^/cm^2^ and grown on membrane inserts (Corning, Corning, NY, USA, cat# 3470) coated with fibronectin. The PCs were combined with ECs (harvested after 2 and 3 passages and seeded at a density of 1 × 10^5^/cm^2^) and grown on the poly-L-lysine–coated inserts. Untreated inserts containing vehicle were used as a control and prepared in the same way as those treated with UT PMN supernatant and NETs. Measurements were obtained following the manufacturer’s protocol. Briefly, electrodes were maintained by soaking the tips once a week in a 1% Tergazyme^®^ solution for 15 min and rinsing with sterile water, a process that was repeated just before disinfection and before beginning an experiment. The STX4 electrodes were disinfected in 70% ethanol for no more than 5 min, followed by rinsing with the medium or phosphate-buffered saline (PBS). This step was followed by the measurement of the resistance in treated samples and controls. 

Cells were allowed to attach and rest for 24 h before the first measurement was performed, and then measurements were made once per day with three measurements per well. First, electrodes were placed in the culture medium for a few minutes. To measure the blank resistance, the electrode was placed in a Transwell^®^ insert without cells filled with cell media. Measurements in experimental wells with cells were then performed. After all measurements, electrodes were disinfected with ethanol, rinsed with sterile water, and allowed to air-dry. To calculate TEER, the surface area of the Transwell^®^ (in cm^2^) was multiplied by the net resistance (the resistance of a blank Transwell^®^ covered by cell culture media subtracted from the measured resistance). 

### 4.7. Permeability Assay

ECs and PCs were cultured on 24-well Transwell^®^ inserts as described above. At 96 h after treatment with UT PMN supernatant and NETs, FITC-conjugated dextran was administered to the upper compartment of the inserts. One hour after the addition of dextran, the fluorescence intensity of the medium in the lower compartments was measured on a fluorescence reader with the excitation at 490 nm and the emission at 520 nm. The 70 kDa FITC-conjugated dextran concentration was calculated with a standard curve. 

### 4.8. Fluorescent Staining

For phalloidin staining, ECs were grown on 4-well fibronectin-coated glass-bottom dishes (Ibidi, Fitchburg, WI, USA, cat#80426). Cells were fixed in 4% paraformaldehyde (Sigma-Aldrich , Burlington, MA, USA, cat# 158127) in PBS (Sigma, cat# P4417), permeabilized with 0.1% Triton X-100 (Sigma, cat# X100) in PBS, and incubated for 1 h at room temperature with Alexa Fluor™ 488 phalloidin (Invitrogen, Waltham, MA, USA, cat# A12379). Samples were washed with PBS and incubated with DAPI for 5 min. The cells were then washed with PBS and mounted on microscope slides using a Fluorescent Mounting Medium (Dako, Glostrup, Denmark, cat# S3023). Images were captured by a Nikon Eclipse Ti2 inverted widefield microscope and processed and analyzed using Fiji-Win 32 software (Version: 2.0.0-rc-49/1.51d). 

### 4.9. Statistical Analysis

Statistical analyses were performed using GraphPad Prism software (Version 10.0.3 (217), San Diego, CA, USA). Multiple groups were compared by one-way or two-way analysis of variance with a Dunn’s and Geisser–Greenhouse correction, respectively; two groups were compared using the Mann–Whitney test with a Welch post-test correction. Data were confirmed to be normally distributed using the Shapiro–Wilk test. *p*-Values of <0.05 were considered significant.

## Data Availability

The data presented in this study are available on request from the corresponding author.
